# Effects of Visual Cortex Activation on the Nociceptive Blink Reflex in Healthy Subjects

**DOI:** 10.1371/journal.pone.0100198

**Published:** 2014-06-17

**Authors:** Simona L. Sava, Victor de Pasqua, Delphine Magis, Jean Schoenen

**Affiliations:** Headache Research Unit, University Department of Neurology, Liège University, Liège, Belgium; University Medical Center Goettingen, Germany

## Abstract

Bright light can cause excessive visual discomfort, referred to as photophobia. The precise mechanisms linking luminance to the trigeminal nociceptive system supposed to mediate this discomfort are not known. To address this issue in healthy human subjects we modulated differentially visual cortex activity by repetitive transcranial magnetic stimulation (rTMS) or flash light stimulation, and studied the effect on supraorbital pain thresholds and the nociceptive-specific blink reflex (nBR). Low frequency rTMS that inhibits the underlying cortex, significantly decreased pain thresholds, increased the 1^st^ nBR block ipsi- and contralaterally and potentiated habituation contralaterally. After high frequency or sham rTMS over the visual cortex, and rMS over the right greater occipital nerve we found no significant change. By contrast, excitatory flash light stimulation increased pain thresholds, decreased the 1^st^ nBR block of ipsi- and contralaterally and increased habituation contralaterally. Our data demonstrate in healthy subjects a functional relation between the visual cortex and the trigeminal nociceptive system, as assessed by the nociceptive blink reflex. The results argue in favour of a top-down inhibitory pathway from the visual areas to trigemino-cervical nociceptors. We postulate that in normal conditions this visuo-trigeminal inhibitory pathway may avoid disturbance of vision by too frequent blinking and that hypoactivity of the visual cortex for pathological reasons may promote headache and photophobia.

## Introduction

“Photophobia” is the clinical term to indicate discomfort to light. It is a common symptom of several neurological and ophthalmological disorders: blepharospasm [1], corneal abrasion, iritis [2], tumors compressing the anterior visual pathways [3], trigeminal neuralgia [4] and, most characteristically, migraine [5].

The pathophysiology of photophobia remains poorly understood.

Reciprocal relations between the visual system and centers involved in trigeminal nociception have been documented in animal studies. Acute exposure to bright light, for instance, increases the number of Fos-like immunoreactive neurons in superficial laminae of trigeminal subnucleus caudalis (Vc/C1) [6] and parasympathetic outflow to the eye [7]. On the one hand, the visual cortex is influenced by projections from the brainstem, especially from dorsal raphe and nucleus raphe magnus [8], [9]. On the other hand, the visual cortex projects downward to brainstem nuclei, including nucleus raphe magnus [10] where it exerts an inhibitory effect [11] and to nucleus cuneiformis [12]. Interestingly, nucleus cuneiformis is part of the descending pain control system and was found hypoactive with fMRI in migraineurs during thermo-nociceptive stimulation [13].

Recently, a novel retino-thalamo-cortical pathway was proposed as a possible anatomo-functional substrate for exacerbation of migraine headache by light. This concept is based on the finding in rat of convergence of retinal afferents and trigeminovascular nociceptive afferents in the posterior and lateral posterior thalamic nuclei [14] whence dural-sensitive thalamic neurons project to various sensory cortical areas including the visual cortex [15]. In humans, MR DTI tractography has revealed a direct connection between optic nerve fibers and the pulvinar [16].

Vanagaite et al. [17] have previously proposed convergence of retinal and trigeminal nociceptive afferents as a possible explanation for photophobia. Direct proof of their hypothesis in humans is still missing, but in a photophobic subject due to corneal irritation by contact lenses, Moulton et al. [13] found light-induced fMRI activation of various structures of the trigeminal nociceptive pathway including thalamus and anterior cingulate cortex. In humans a reciprocal relation between visual input and trigeminal nociception is suggested by the decreased tolerance to light after painful stimulation of the ophthalmic branch of the trigeminal nerve [18] and the reduction of trigeminal pain thresholds after light stimulation in migraine patients [19], [20]. In a PET study, continuous light stimulation induced a stronger activation of the visual cortex in migraine subjects than in healthy subjects, and, when it was combined with a painful stimulation in the trigeminal territory, the activation was markedly greater in migraine patients [21].

The aim of our study was to testing healthy volunteers the hypothesis that the visual cortex is able to modulate excitability in the trigeminal nociceptive system, which would be relevant for migraine-related photophobia and for migraine headache. As indices for excitability in the trigeminal sensory system we have chosen sensory and pain detection thresholds to supraorbital electrical stimuli as well as amplitude of the nociceptive-specific blink reflex (nBR), a brain stem reflex modified by cortical and subcortical afferents [22], [23], [24], [25]. To modulate the visual cortex, we used flash light stimulation or repetitive transcranial magnetic stimulation (rTMS) at high or low stimulation frequency [26], [27]. As controls, we applied sham rTMS over the visual cortex and effective repetitive magnetic stimulation (rMS) over the greater occipital nerve.

## Materials and Methods

### Ethics Statement

The project was reviewed and approved by the Ethic Committee of the CHR Citadelle Hospital, Faculty of Medicine, University of Liège, Belgium, and was conform to the Declaration of Helsinki. All participants gave written informed consent prior to testing. 2 participants of 14 and 16 years old were included in our study, a written informed consent was given by their parents.

### Subjects

The experiments were performed on 63 healthy subjects (HS) without a personal or family history of primary headache. We applied rTMS on the visual cortex, at low and high frequency, in 21 subjects (12 females, 9 males, mean age 25.9±8.03) and flash light stimulation in 22 subjects (12 females, 10 males, mean age 26.59±9.29). As controls for rTMS, we used occipital sham stimulation in 13 subjects (8 females, 5 males, mean age 25.38±11.18) and effective stimulation over the greater occipital nerve in 7 subjects (5 females, 2 males, mean age 29±10.59). As recommended for rTMS [28], all subjects were devoid of any medical condition and had no personal or family history of epilepsy. To avoid interference with changes of cortical excitability due to hormonal variations, females were recorded during mid-cycle. All subjects were naïve for rTMS.

### Nociceptive Blink Reflex

Subjects were seated relaxed in a comfortable armchair in an illuminated room and were asked to leave their eyes open. The nociceptive-specific blink reflex was elicited according to the method described by others [29], [30], before and immediately after the rTMS session or flash light stimulation.

We used a custom-made planar concentric electrode (central cathode: 1 mm D; insert: 8 mm; anode: 23 mm OD) placed on the forehead close to the supraorbital foramen on the right side. The concentric electrode has the advantage of exciting preferentially Aδ fibers [29], [20], [31], [32], but at the same time C-fibers and Aβ fibers may also be recruited [33]. It seems that the recruitment of Aβ fibers may vary with regard to the site of stimulation, stimulus repetition rate and duration as well as penetration of the electrode in the skin [34].

Recording electrodes were placed below the orbit (active) over the orbicularis oculi muscle and lateral to the orbit (reference) on both sides. A ground electrode was placed on the root of the nose. The signal was recorded with a sampling rate of 5000 Hz and sweep duration of 150 ms (1401, Signal Averager, Cambridge Electronic Design).

We first determined perception and pain thresholds by using ascending and descending sequences of 0.2 mA intensity steps. The mean number of assessments per participant was 11±4 for sensory thresholds and 15±8 for pain thresholds. The electrical stimuli consisted of monopolar square pulses with 0.2 ms duration. To elicit the nBR, the final stimulus intensity was set at 1.5 times the initial individual pain threshold. Interstimulus intervals varied pseudo-randomly between 15 and 17 s. We recorded 16 rectified EMG responses that were averaged off-line. As previously described, the first response of each nBR recording session was excluded from the signal analysis to avoid contamination with startle responses [30], [31], [32]. The remaining 15 sweeps were averaged in 3 sequential blocks of 5 responses. For each averaged block, amplitude of the R2 reflex was expressed as its area under the curve (AUC). To minimize R2 AUC variability due to inter-individual threshold differences we used the ratio between the area and the square of the stimulus intensity (AUC/i^2^) as an index of nBR amplitude changes, as recommended by Sandrini et al. [35]. Habituation of the nBR R2 was defined as the percentage change of the R2 area between the 1st and the 3rd block of averages.

### Magnetic Stimulation

#### rTMS over the visual cortex

We used a Magstim Rapid magnetic stimulator (Magstim Co. Ltd, Whitland, Dyfed, UK), connected to a 2×7.0 cm figure-of-eight coil, with a maximal stimulator output of 1.2 T. Using single pulses, we first identified the phosphene threshold, defined as the lowest stimulation intensity (expressed as a percentage of the maximal stimulator output) able to evoke phosphenes in at least three out of five pulses [36]. The coil was placed in a vertical position (its handle pointing upward) on the inion-nasion line, with its inferior limit 1 cm above the inion. Stimulation was applied initially at 30% of stimulator output. The intensity of the stimulation was increased by 2% steps until the subject reported phosphenes. Increasing and decreasing the intensity in 1% steps then refined the threshold. In participants who did not report phosphenes at the 100% intensity level, the procedure was repeated with the coil placed 1 or 2 cm higher or lower and, if necessary, to the right or to the left, before accepting the absence of phosphenes. In this case, we placed the coil over the left motor area and determined the motor threshold. In accordance with recommended safety guidelines [28], stimulus intensity was set to the phosphene threshold (PT) or to 110% of the motor threshold, if no phosphenes were elicited.

We used two different stimulation frequencies in a randomised order: 1 Hz (low frequency rTMS) and 10 Hz (high frequency rTMS) with at least a 24 hour-interval between the 2 sessions, as recommended by others for stimulation of the motor cortex [37]. 1 Hz rTMS was applied in a single train without interruption for 15 minutes. 10 Hz rTMS was applied in 20 trains of 40 pulses with inter-train intervals of 10 seconds. For both frequencies, a same amount of 800 pulses was thus delivered.

### Sham rTMS Over the Visual Cortex

In 13 subjects blinded to the stimulation protocol, 10 Hz rTMS sham stimulation was delivered with the coil placed at a 90° angle to the occipital region, with its anterior border pressed against the scalp. The rTMS intensity was fixed at the intensity of the phosphene threshold or 110% of the motor threshold. Twenty trains of 40 pulses with an inter-train interval of 10 seconds were delivered for 5 minutes. In the sham situation, there is an acoustic perception of the stimulation, but no brain activation occurs [38]. We decided to enrol only subjects completely naive to rTMS in order to ensure blinding.

### rMS Over the Greater Occipital Nerve (GON)

We performed 1 Hz and 10 Hz rMS over the right GON in 7 HS by placing the figure-of-eight coil over the emergence of the GON just beneath the superior nuchal line. We considered as optimal the location where the sensation induced by the magnetic pulse radiated to the parietal region of the head. The rMS intensity was fixed at the phosphene threshold or 110% of the motor threshold found during the previous session of effective rTMS, to make a comparable control protocol. The patterns of 1 Hz or 10 Hz stimulation were the same as those applied over the visual cortex.

### Flash Light Stimulation

We used the Microflash MF 9607178 stimulator (Micromed & Co., Mogliano Veneto, IT) for flash light stimulation in 22 subjects. We placed the light stimulator in front of the subjects at a 15 cm distance, asking them to look at the stimulator during the whole session. The stimulation was at 27.8 lux (0.63 cd). To minimize attenuation of light perception due to continuous stimulation without spatial or temporal contrast [39], [40], flash frequency was set at 8 Hz for 4 minutes in a quiet room with dimmed light.

### Data Processing and Statistical Analysis

STATISTICA for Windows version 8.0 (StatSoft, Inc. Tulsa, OK, USA) was used for all statistical analyses. Wilcoxon’s test was applied to compare the differences between pre- and post-stimulation in perception and pain thresholds, AUC of the 1^st^nBR block and slope of amplitude changes over 3 consecutive blocks of nBR averagings, ipsilaterally and contralaterally. Mann-Whitney’s test was used to compare the differences between stimulation methods. Spearman’s test was used for the correlation analysis. All results were considered significant at the 5% level (p<0.05).

## Results

### 

#### Transcranial magnetic stimulation – visual cortex

12 participants out of 21 (57.14%, 3 males and 12 females) stimulated with TMS over the visual cortex reported phosphenes. The phosphene threshold (expressed as a percentage of the maximal stimulator output) was 66±4.7%. The motor threshold was determined in the remaining 9 participants (42.86%, 7 males and 2 females) and was 58±8% of the maximal stimulator output. We observed a significant relation between the presence of phosphenes and female gender (*p = 0.04*). There was no correlation between intensity of rTMS and the effect on the nBR. After 1 Hz rTMS over the visual cortex, the supraorbital pain threshold was significantly decreased (*p = 0.001)* (Table 1), while the sensory threshold remained unchanged.

**Table 1:** Means of electrophysiological data.

	Number	Age		ST	PT	AUC 1°block ipsilateral	AUC 1° block contralateral
**rTMS 1 Hz visual cortex**	21	27.45±10.68	before	0.67±0.19	5.85±2.28	0.027±0.034	0.019±0.024
			after	0.71±0.19	4.69±2.58	0.031±0.033	0.025±0.027
			p	0.17	***0.001***	***0.024***	***0.036***
**rTMS 10 Hz visual cortex**	21	27.45±10.68	before	0.67±0.23	5.61±2.52	0.031±0.042	0.026±0.038
			after	0.72±0.23	5.64±3.21	0.023±0.023	0.015±0.015
			p	0.71	0.07	0.32	0.1
**rTMS Sham**	13	25.38±11.18	before	0.89±0.31	4.79±2.63	0.034±0.070	0.037±0.0889
			after	0.79±0.24	4.98±2.65	0.014±0.020	0.012±0.019
			p	0.06	0.47	0.19	0.27
**rTMS 1 Hz Right GON**	7	29±10.59	before	0.72±0.18	4.4±1.88	0.03±0.048	0.027±0.041
			after	0.90±0.16	4.73±2.1	0.031±0.045	0.037±0.056
			p	0.12	0.23	0.49	0.31
**rTMS 10 Hz Right GON**	7	29±10.59	before	0.74±0.25	4.78±2.8	0.037±0.052	0.036±0.056
			after	0.73±0.26	4.71±1.82	0.02±0.018	0.020±0.027
			p	0.83	0.61	0.17	0.31
**Flash light**	22	26.60±9.30	before	0.93±0.19	5.39±2.82	0.022±0.022	0.013±0.011
			after	1.0±0.17	6.11±2.69	0.009±0.010	0.008±0.008
			p	0.41	***0.008***	***0.004***	***0.001***

rTMS: repetitive transcranial magnetic stimulation; ST: sensory threshold; PT: pain threshold.

**Figure 1:**
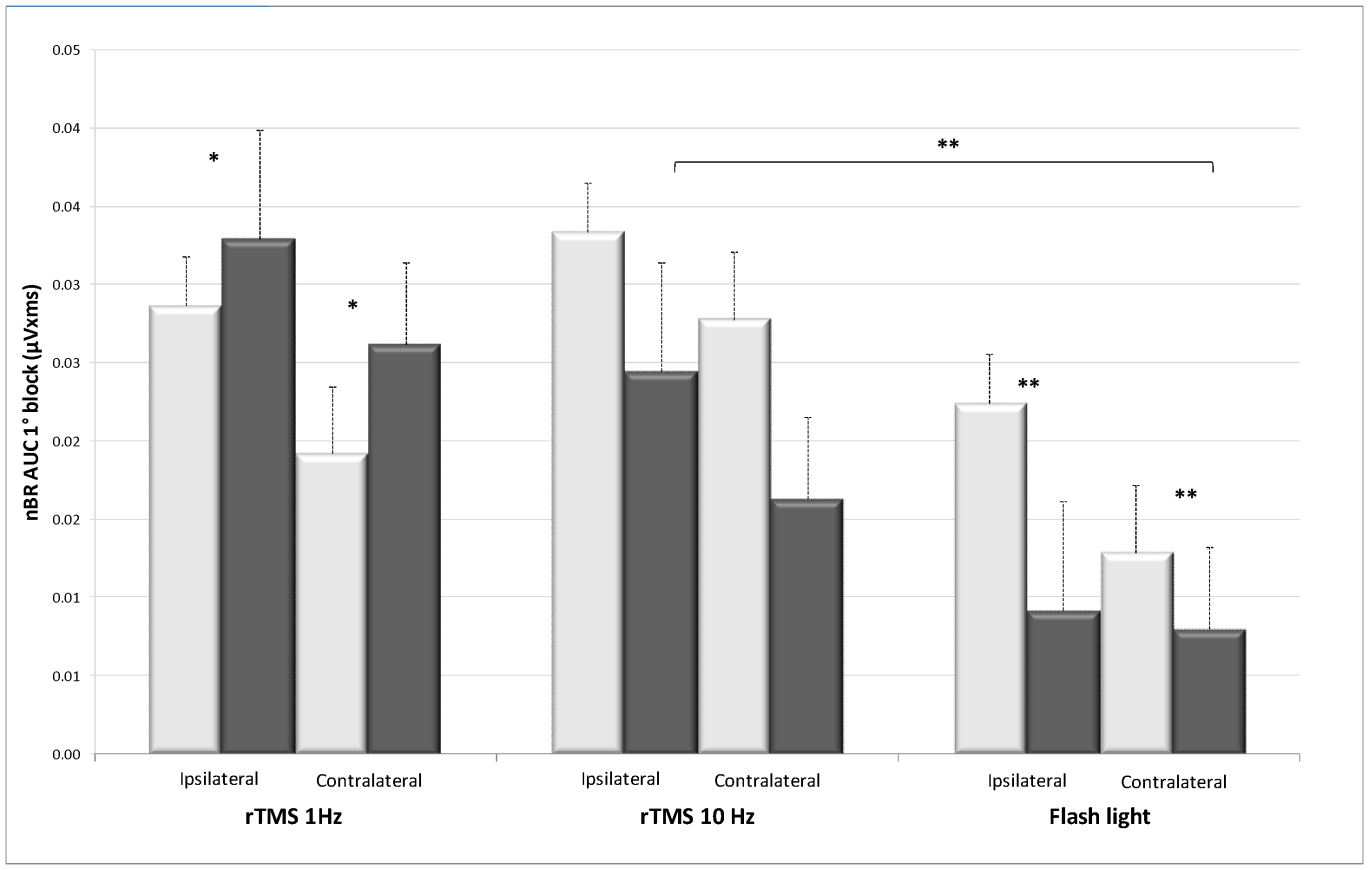
First block of 5 ispilateral and contralateral nBR responses (area under the curve in µVxms ± sem) before (light bars) and after (dark bars) 1 Hz rTMS, 10 Hz rTMS over the visual cortex, or flash light stimulation. ** p<0.01; * p<0.05. The inhibitory effect on the nBR is significantly stronger after flash light stimulation than after10 Hz rTMS over the visual cortex.

**Figure 2:**
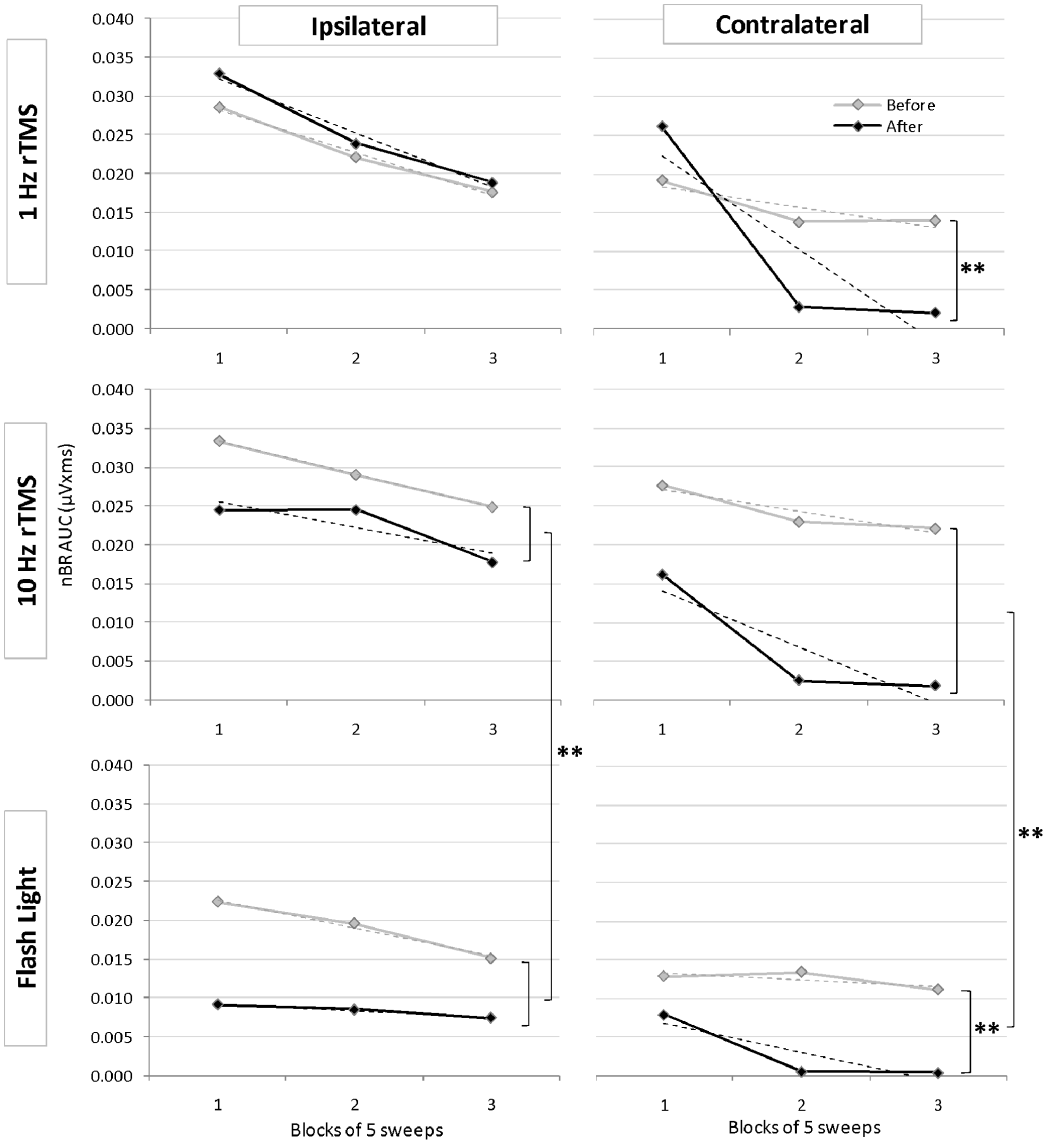
Area under the curve of ipsi- and contralateral nociceptive blink reflexes in 3 successive blocks of 5 averaged responses before (grey lines) and after (black lines) 1 Hz rTMS, 10 Hz rTMS over the visual cortex, or flash light stimulation. Vertical brackets indicate significant differences before and after stimulation, or between stimulation modalities. ** p<0.01; * p<0.05.

Moreover, 1 Hz rTMS significantly increased amplitude of the 1^st^ nBR block expressed as AUC/i^2^ both ipsi- and contralaterally to the supraorbital stimulation (*p = 0.024* and *p = 0.036* respectively) (Table 1, Fig. 1). By contrast, habituation was significantly potentiated contralaterally to the stimulated side (*p = 0.0002*) (Fig. 2).

We found no significant variation of sensation or pain thresholds, nBR amplitude and habituation after the 10 Hz rTMS session (Fig. 2) or after sham rTMS.

#### Magnetic stimulation – right GON

There was no significant change of sensory thresholds, nBR amplitude or habituation after stimulating the right GON, neither for 1 Hz rMS, nor for 10 Hz rMS (Table 1).

#### Photic stimulation

**Figure 3:**
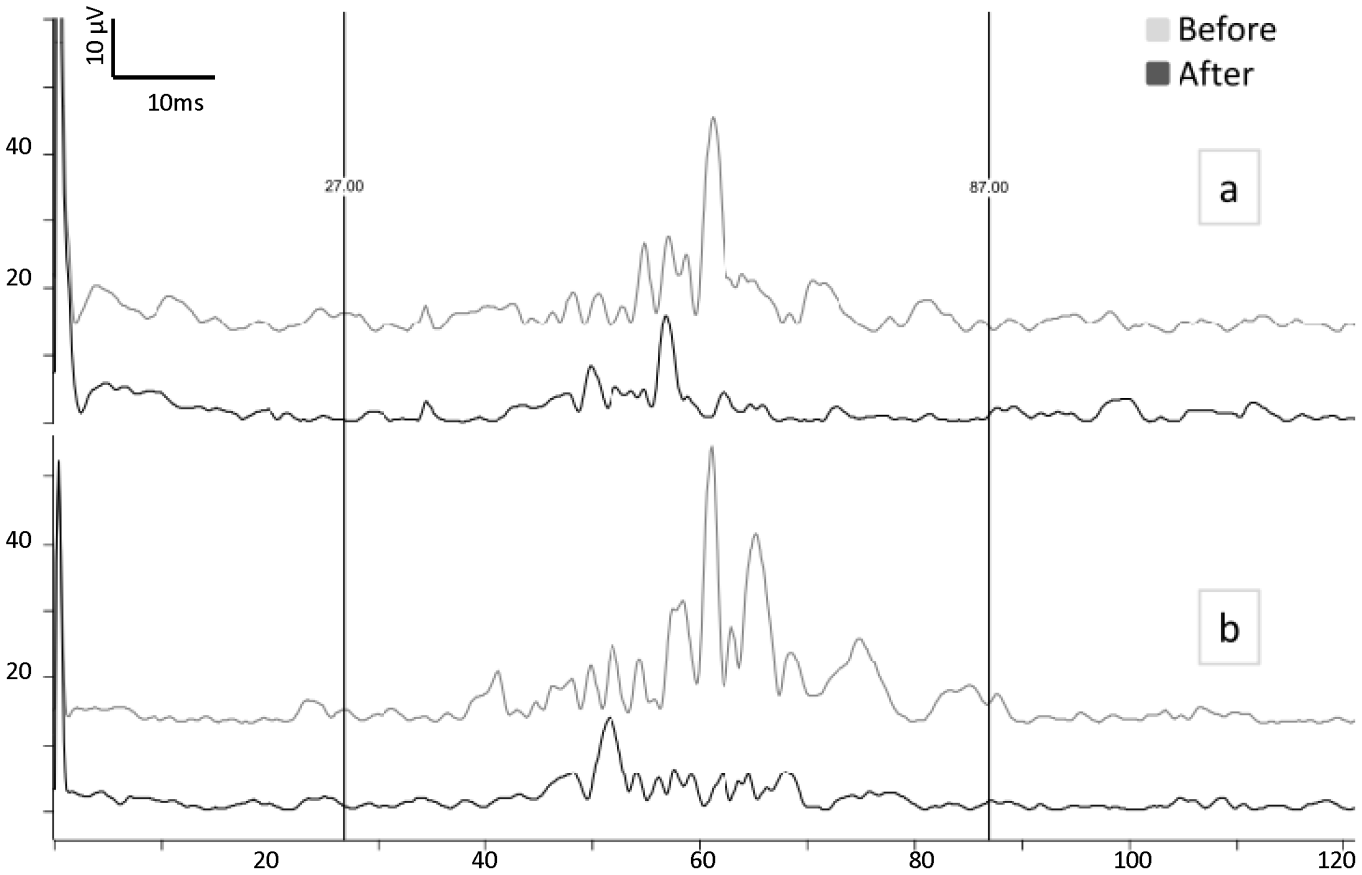
Averaged ipsi- (a) and contralateral (b) nociceptive blink reflex (rectified EMG) in a subject before (grey trace) and after (black trace) flash light stimulation.

Figure 3 shows an illustrative recording of the nBR responses before and after flash light stimulation. The latter increased pain threshold (*p = 0.008*) (Table 1), decreased AUC/i^2^ of the 1^st^nBR block (*p = 0.004* ipsilateral; *p = 0.001* contralateral) and increased habituation contralaterally (*p = 0.002*) (Fig. 1 and Fig. 2). Although both 10 Hz rTMS and flash light stimulation are known to activate the visual cortex, the effect on the nBR was significantly more pronounced after flash stimulation than after excitatory rTMS. This was the case in particular for ipsilateral (*p = 0.002*) and contralateral (*p = 0.027*) 1^st^ nBR blocks and even more so for increase in habituation of ipsilateral (*p = 0.00008)* and contralateral responses (*p = 0.00000*) (Fig. 2).

## Discussion

Our data add to the existent literature experimental evidence in humans for a functional connection between the visual cortex and 2^nd^order nociceptors in spinal trigeminal nucleus.

As an objective marker of excitability in the trigeminal nociceptive system, we have chosen the nociceptive specific blink reflex (nBR). Ophthalmic nerve afferents, mainly Aδ fibers, mediate the R2 response and reach via the ponto-medullary descending spinal trigeminal tract wide dynamic range 2^nd^ order nociceptors in caudal spinal trigeminal nucleus whence impulses ascend to the facial nuclei in the pons via a bilateral trigemino-facial pathway located in the lateral tegmental field [22], [23], [29], [30].

We have found that sensation and pain thresholds of the supraorbital electrical stimulus as well as area under the curve (AUC) and habituation of the nBR are modulated differentially by excitatory or inhibitory repetitive transcranial magnetic stimulations (rTMS) over the visual cortex and by flash light stimulation. As controls for visual cortex rTMS, we used sham rTMS and repetitive magnetic stimulation (rMS) over the right greater occipital nerve (GON).

As can be seen from figure 2, habituation of the contralateral R2 response increases in our study whatever the experimental intervention is. During repeated stimulation with an inter-stimulus interval of 15–17sec as used here, nBR responses clearly habituate bilaterally in healthy subjects, but not in migraine patients [31]. The more pronounced habituation of contralateral responses could be related to the fact that 1^st^ block amplitude is overall lower on the side opposite to the supraorbital stimulus, a relation that was also reported for visual evoked potentials [32].

We will discuss the changes induced by modulating visual cortex activity and thereafter the possible relevance of our findings for migraine pathophysiology.

### Modulations of Visual Cortex Activity

The supraorbital pain threshold decreased after 1 Hz rTMS over the visual cortex but increased after flash light stimulation. Concordantly, amplitude of the 1^st^ block of five averaged nBR responses increased bilaterally after the former and decreased after the latter. By contrast, 10 Hz rTMS over the visual cortex produced no significant changes, but it was followed by a numerical decrease of pain sensitivity and nBR amplitude. Taken together, these results may suggest that the visual cortex exerts at baseline a sustained top-down inhibitory effect on trigeminal nociception. Indeed rTMS at low frequency is supposed to inhibit the underlying cortex [26] while the flash stimulation excites visual areas. This is in line with a study showing in healthy volunteers a tendency for an increase of pain perception thresholds in the innervation territories of the trigeminal and greater occipital nerves after intense light stimulation [20]. We have found a similar difference between low and high frequency rTMS over the visual cortex in a study of visual evoked potentials (VEP) in healthy subjects: 1 Hz rTMS reduced amplitude of the 1^st^ VEP block, while 10 Hz rTMS had no effect [41]. As a possible explanation for these differential results, we postulated that in normal subjects the cortical baseline activation level is close to the “ceiling”, i.e. the upper level of the cortical activation range, hence it cannot be further activated by the excitatory 10 Hz rTMS but it can be decreased by the inhibitory 1 Hz rTMS. This explanation is supported *a contrario* by the finding that in migraine patients who may have a lowered cortical baseline activation level of the visual cortex and a decrease in 1^st^ block VEP amplitude at baseline, 10 Hz rTMS increases 1^st^ block VEP amplitude whereas 1 Hz has no effect [36]. The difference between 10 Hz rTMS and flash light stimulation in the present study is likely due to the fact that the former moderately increases the activation level of the visual cortex while the latter activates more robustly the visual areas via the retino-geniculo-cortical pathway of visual perception.

Extrageniculate visual pathways may provide an alternative explanation. In cat and monkey there is evidence for a pathway connecting the retina with the visual cortex via the pulvinar [42], [43]. More recently, Noseda et al. [14] have demonstrated in animals projections from retinal ganglion cells to the posterior thalamus, whence via caudate-putamen and external capsule they reach multiple cortical regions, including the binocular area of the primary visual cortex. The authors suggest that this novel pathway may explain why even blind migraine patients experience photophobia. One may hypothesize that these extrageniculate pathways, if they exist also in humans, can induce an inhibitory top-down modulation of trigeminal nociceptors by thalamic neurons after flashing light but not after direct electro-magnetic activation of the visual cortex.

In migraine patients the photophobia threshold is lower than in healthy subjects after a painful stimulation applied on the forehead [18]. Along the same line, continuous light was shown to produce detectable oxygenation changes in the visual cortex of healthy subjects, only if combined with painful heat stimulation in the territory of the ophthalmic nerve [21]. The authors explain their finding by a “bottom-up” activation by the trigeminal nociceptive stimuli of visual areas rendering them responsive to a stimulus that normally produces no detectable activation because of its continuous nature and absence of any contrast pattern. Activation of visual areas by pain may not be specific to the trigeminal system, as it has also been found after pain applied to the hand [44], [45]. In our study we assume that the cortical activation by the flickering light stimulation was sufficient to unravel an opposite “top-down” inhibitory control by the visual cortex of nociceptive trigeminal processing.

Sensory terminals of the greater occipital nerve are interposed between the coil of the magnetic stimulator and the occipital cortex. The electro-magnetic pulses could activate some of these peripheral neural structures and produce an afferent input that may at least in part reach the spinal trigeminal nucleus and modify its excitability. To exclude this possibility, we have positioned the coil over the greater occipital nerve underneath the upper nuchal line in control experiments. Magnetic stimuli over the GON had no significant effect on the nBR, which suggests that putative activation of peripheral afferents is not a confounding factor in our rTMS results.

Gender may be a confounding factor in activation studies of the visual cortex. Magnetophosphenes are indeed more prevalent in females than in males in our study. A sexual dimorphism of magnetophosphenes was not studied or reported in previous studies. Such dimorphism is present in migraine and sex hormones are well known to modulate cortical excitability in humans and in animals [46]. The magnetic stimulation intensity to evoke phosphenes in our study is in line with that found in other studies [36]. In our study we did not use phosphene thresholds after rTMS to verify changes in excitability for several reasons. First, it is well established that rTMS is able to modify visual cortex excitability as indexed by visual evoked potentials (VEP) [41]. Second, although magnetophosphenes are easy to use as indicators of visual cortex excitability, they are not very reproducible and less reliable than VEPs [47]. Unfortunately, because of the design of the experimental protocol and the necessity to record blink reflexes as soon as possible after rTMS or flash stimulation, there was no sufficient time for VEP recordings.

Another confounding factor in our study could be a change in excitability of the facial nucleus motor neurons that contract orbicularis oculi muscles. Although we cannot exclude this possibility, it is highly unlikely to have influenced our results significantly as the decrease of nBR amplitude was associated with an increase in pain thresholds after flashing light.

### Possible Physiological and Pathophysiological Relevance

The top-down relation between the visual cortex and the trigeminal system may play a role in the pathophysiology of photophobia. In rodents bright light is able to activate neurons at multiple sites of the trigemino-cervical complex [6], which is associated with activity of the olivary pretectal nucleus and the superior salivary nucleus [7]. Given its role in saccades and blink [48], the superior colliculus is a possible relay for the effects we have observed. It receives indeed projections from the visual cortex [49] as well reticular and cervical spinal cord projections involved in eyelid movements during the blink reflex [50].

The top-down control we have shown here differs from the one reported in cats by Lambert et al. [11]. These authors found that cortical spreading depression (CSD) or light flash inhibits activity of neurons in nucleus raphe magnus (NRM) and hence disinhibits the responses of trigeminal nociceptors receiving dural input. Multiple waves of CSD antagonized the inhibitory effect of NRM stimulation on responses of trigeminal neurons to dural but not to skin mechanical stimulation. The apparent discrepancy between Lambert et al’s [11] and our results may have several explanations. First, there are obvious methodological differences. Lambert et al. [11] used extracellular recordings in trigeminal nucleus caudalis as opposed to indirect assessment of the excitability of trigeminal neurons interposed in the nBR circuit in our study. Ten Hz flash light stimulation was applied for 10 minutes in the cats, while 8 Hz flashes were delivered for 4 minutes to our subjects. Moreover, species differences in visuo-trigeminal interactions cannot be excluded considering the differences in vision between cats and humans. Lastly, CSD, albeit starting with a brief depolarization of cortical neurons, chiefly induces a long-lasting depression of neuronal activity. If one accepts that such a depression might have similar effects on the visual cortex and its connectivity as inhibitory 1 Hz rTMS, both the study in cat and ours in humans would concord in showing that the visual cortex exerts a tonic descending inhibitory action on trigeminal nociceptors.

An inhibitory top-down control by the visual cortex of the trigeminal nociceptive system may have other implications in health and disease. In normal conditions it could contribute to avoid excessive blinking during visual attention. Viewing the stimulated site can decrease pain perception in peripheral limbs of healthy subjects, a phenomenon called “visual analgesia” [51], [52], [53]. In functional MRI studies, the top-down inhibitory effect of vision on laser-heat evoked pain in the hand is associated with diminished activation in somatosensory cortex SI and operculoinsular cortex but not in anterior cingulate cortex [52]. Our study would be in line with a similar effect of vision in the nociceptive trigeminal system, although a similar analgesic effect in the trigeminal territory by viewing the face remains to be demonstrated.

Tonic inhibition of trigeminal nociceptors by the visual cortex could also be relevant for the pathophysiology of the migraine headache. We have shown that between attacks most migraineurs are characterized by lack of habituation of VEPs [54] resulting in greater net activation of the visual cortex during repetitive stimulation (hyper-responsivity). By contrast VEP habituation normalizes just before and during the migraine attack [55] as well as in chronic migraine [26], [56], which reduces net activation of the visual cortex. If our present findings are applied to the changes in cortical activity over the migraine cycle, the trigeminal nociceptive system would be rather inhibited at a distance from an attack because of visual cortex hyper-responsivity, while it would be disinhibited just before and during the attack as well as in chronic migraine because of a decrease in cortical responsivity. The finding in migraine patients of a deficient habituation of the nBR in the interictal period and its normalization ictally [29], [30], [32] favours such an excitability cycle of trigeminal nociceptors, as habituation is inversely related to amplitude of the 1^st^ block of responses and thus to baseline excitability.

In addition, the migraine aura is caused by CSD that, as mentioned above, comprises an initial brief neuronal depolarization front, followed by a long-lasting depolarization block of neuronal activity in the visual cortex. Applying our results to the migraine aura, the long-lasting inhibition may cause disinhibition of trigeminal nociceptors and contribute to the CSD-induced neuronal activation in trigeminal nucleus caudalis [57] and thus to the migraine headache.

## Conclusion

Our study demonstrates in healthy subjects a functional relation between the visual cortex and the trigeminal nociceptive system, as assessed by the nociceptive blink reflex. Our results favour of a top-down inhibitory pathway from the visual areas to trigemino-cervical nociceptors. This pathway may be functionally different from the one attributing to the visual cortex a disinhibitory role on nucleus raphe magnus-mediated inhibition of dural trigeminal nociceptors in cats. In normal conditions the top-down inhibitory pathway may avoid that too intensive blinking disturbs vision. In case of increased responsivity of the visual cortex, like during the interictal period in migraine, the visuo-trigeminal inhibitory pathway may reduce trigeminal nociception. By contrast, when visual cortex responsivity is decreased like during the migraine attack, or in chronic migraine, reduced activation of the visuo-trigeminal inhibitory pathway may increase excitability of trigeminal nociceptors and hence favour headache.
