# Correction: Effects of Visual Cortex Activation on the Nociceptive Blink Reflex in Healthy Subjects

**DOI:** 10.1371/journal.pone.0112036

**Published:** 2014-10-22

**Authors:** The PLOS ONE Staff

The third and fourth authors, Delphine Magis (DM) and Jean Schoenen (JS), should be noted as contributing equally to this work.
